# Effect of Tryptophan-Derived AhR Ligands, Kynurenine, Kynurenic Acid and FICZ, on Proliferation, Cell Cycle Regulation and Cell Death of Melanoma Cells—In Vitro Studies

**DOI:** 10.3390/ijms21217946

**Published:** 2020-10-26

**Authors:** Katarzyna Walczak, Ewa Langner, Anna Makuch-Kocka, Monika Szelest, Karolina Szalast, Sebastian Marciniak, Tomasz Plech

**Affiliations:** 1 Department of Pharmacology, Medical University of Lublin, Chodźki 4a, 20-093 Lublin, Poland; ewa.langner@umlub.pl (E.L.); anna.makuch@umlub.pl (A.M.-K.); m.wlodarczyk214@gmail.com (M.S.); karolina.szalast@umlub.pl (K.S.); sebastian.marciniak@umlub.pl (S.M.); tomasz.plech@umlub.pl (T.P.); 2 Department of Medical Biology, Institute of Rural Health, Jaczewskiego 2, 20-090 Lublin, Poland

**Keywords:** tryptophan, kynurenine, kynurenic acid, FICZ, AhR, melanoma, proliferation, cell death

## Abstract

Tryptophan metabolites: kynurenine (KYN), kynurenic acid (KYNA) and 6-formylindolo[3,2-b]carbazole (FICZ) are considered aryl hydrocarbon receptor (AhR) ligands. AhR is mainly expressed in barrier tissues, including skin, and is involved in various physiological and pathological processes in skin. We studied the effect of KYN, KYNA and FICZ on melanocyte and melanoma A375 and RPMI7951 cell toxicity, proliferation and cell death. KYN and FICZ inhibited DNA synthesis in both melanoma cell lines, but RPMI7951 cells were more resistant to pharmacological treatment. Tested compounds were toxic to melanoma cells but not to normal human adult melanocytes. Changes in the protein level of cyclin D1, CDK4 and retinoblastoma tumor suppressor protein (Rb) phosphorylation revealed different mechanisms of action of individual AhR ligands. Importantly, all tryptophan metabolites induced necrosis, but only KYNA and FICZ promoted apoptosis in melanoma A375 cells. This effect was not observed in RPMI7951 cells. KYN, KYNA and FICZ in higher concentrations inhibited the protein level of AhR but did not affect the gene expression. To conclude, despite belonging to the group of AhR ligands, KYN, KYNA and FICZ exerted different effects on proliferation, toxicity and induction of cell death in melanoma cells in vitro.

## 1. Introduction

The skin is constantly exposed to various substances with pro- and anti-carcinogenic potential. Environmental pollution, skin care products and UV radiation can affect various processes in the skin. Environmental UV exposure, fair color of skin and hair, family history of melanoma and a high number of melanocytic nevi are well-characterized risk factors for developing melanoma [1]. Melanoma is rare among all types of skin cancers (less than 5%) but the most aggressive [2,3]. A median survival rate of patients with metastatic melanoma is approximately 6 months [2]. A poor prognosis for patients with advanced melanoma results from rapid metastasis, resistance to anti-cancer therapies and immunosuppressive abilities. Thus, there are two main strategies in melanoma prevention and treatment: inhibition of melanocyte-to-melanoma transition and inhibition of melanoma metastasis.

The aryl hydrocarbon receptor (AhR) is a major sensor of chemical signals and is mainly expressed in barrier tissues (including skin, liver, lungs and the gastrointestinal tract) which may be exposed to environmental factors [4]. AhR, a ligand-activated transcription factor, is a member of the evolutionarily conserved family of per-arnt-sim basic helix-loop-helix transcription factors (PAS-bHLH). AhR has been mainly associated with dioxin toxicity and detoxification of xenobiotic compounds, but it also plays a role in several physiological and pathological processes in skin such as detoxification, cellular homeostasis, skin pigmentation, skin immunity and carcinogenesis [5,6]. The role of AhR in melanomagenesis is not clear, however, previous studies revealed involvement of this receptor in melanoma cell dedifferentiation and metastases in response to inflammation [7].

Some tryptophan-derived metabolites including kynurenine (KYN), kynurenic acid (KYNA) and 6-formylindolo[3,2-b]carbazole (FICZ) are considered as AhR ligands [8,9]. Tryptophan, an essential amino acid, is well-known to be the most potent near-UV absorbing chromophore. Several tryptophan metabolites have various biological activities and may potentially affect processes in skin. However, the effect of KYN, KYNA and FICZ on melanomagenesis and melanoma progression has not been fully investigated.

KYN, an endogenous agonist of AhR, is produced enzymatically from tryptophan by indoleamine 2,3-dioxygenase (IDO) and tryptophan 2,3-dioxygenase (TDO) [10]. Previous studies revealed that both enzymes are expressed and upregulated in various cancer types, including melanoma [11,12]. The direct activity of KYN on cancer cell proliferation has not been fully studied, but it is supposed that this tryptophan metabolite plays a crucial role in the antitumor immune response. Opitz et al. revealed that KYN is produced during brain cancer progression and inflammation in the local microenvironment in amounts sufficient to activate the human AhR, suppressing antitumor immune responses and promoting cancer cell survival and motility [13]. Similarly, KYN induced an antiapoptotic response in breast cancer cells [14].

On the other hand, previous studies revealed antiproliferative and antimigratory properties of KYNA against various types of cancer cells [15]. However, its role in cellular processes in melanoma has not been revealed. KYNA, a natural ligand for AhR, is enzymatically formed from KYN and is present in almost all human body fluids and tissues [15]. Additionally, KYNA may be absorbed from the gastrointestinal tract due to its presence in many food products [16,17,18]. The role of KYNA in skin physiology and pathology has not been fully studied; however, phototoxic effects of KYNA on erythrocytes and glia cells have been reported [19,20]. Importantly, KYN and KYNA may be enzymatically synthesized by skin cells [21].

FICZ is a tryptophan photometabolite formed by exposure to UV or visible irradiation [22]. It plays a crucial role in several physiological processes including skin homeostasis, regulation of skin immunity and circadian rhythm, genomic stability and response to UV exposure [23]. FICZ is also considered to be the most potent UVA photosensitizer, leading to induction of oxidative stress and oxidative DNA lesions, which consequently leads to cell death of keratinocytes [23]. Some previous studies confirmed the potential role of FICZ in the differentiation-induction therapy of leukaemia [24]; however, its contribution to melanomagenesis has not been fully revealed. Mengoni et al., in in vitro studies, reported that FICZ is involved in inflammation-induced dedifferentiation [7]. On the other hand, FICZ is also considered as a sensitizer of photooxidative stress, leading to photodynamic elimination of skin cancer cells in vitro and in vivo [25].

There are three potential interactions between tryptophan-derived AhR ligands and skin cells: (1) direct contact or topical application on the skin surface, as they are present in several herbs, honey-bee products used in beauty and body treatments, and body care products [16,18,26], (2) endogenous production in skin cells, and (3) via the food chain (as KYNA is present in some food products [16,18]). Taking into consideration the continuous external and internal exposure of skin cells to the tryptophan-derived AhR ligands and the ambiguous role of AhR in melanomagenesis and melanoma progression, determining the negative or positive role of tested substances in carcinogenesis seems to be a priority. The aim of the study was to determine the biological activity of the selected tryptophan-derived AhR ligands on melanomagenesis by investigating the effect of KYN, KYNA and FICZ on the proliferation, cytotoxicity and cell death of normal human melanocytes and melanoma cells in vitro.

## 2. Results

**Figure 1:**
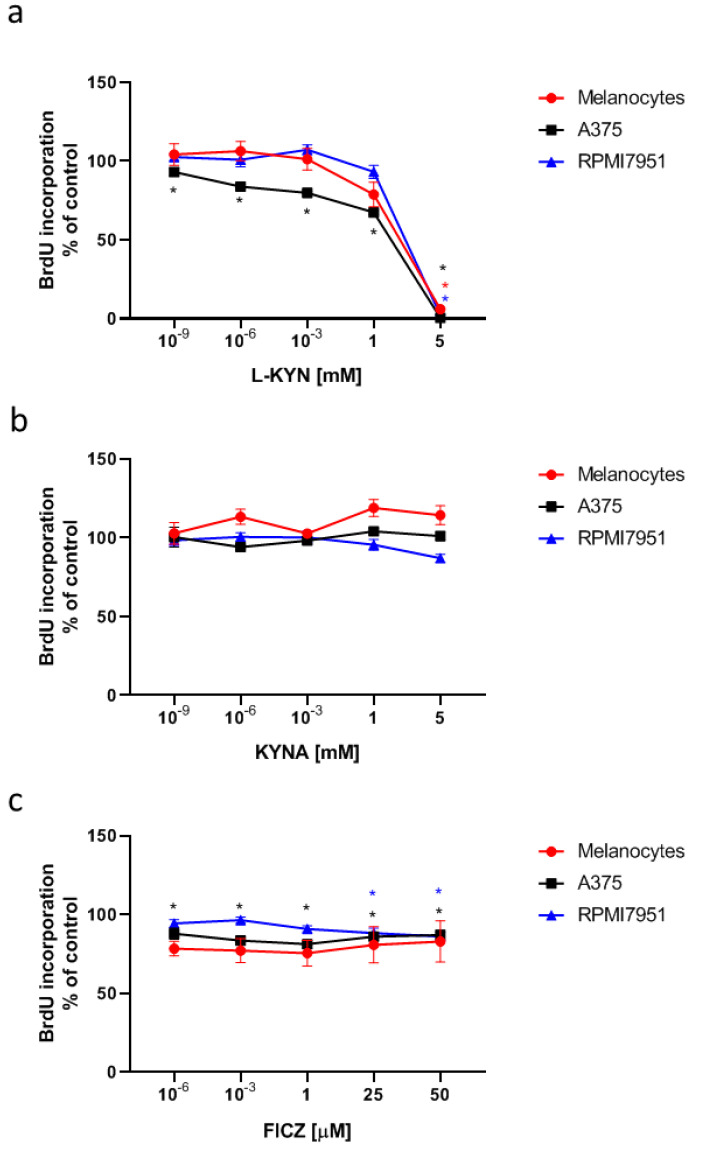
The effect of L-KYN (**a**), KYNA (**b**) and FICZ (**c**) on proliferation (DNA synthesis) of melanocytes and melanoma A375 and RPMI7951 cells. Normal human adult primary epidermal melanocytes (HEMa) and human melanoma A375 and RPMI7951 cells were exposed to fresh medium (control, C) or serial dilutions of L-KYN, KYNA and FICZ for 24 h. The effect of tested compounds on proliferation (DNA synthesis) was determined by means of BrdU Assay. Data represent a mean value (% of control; C = 100%) ± SEM of eight independent experiments. Values significant (*) in comparison to control (100%) with *p* < 0.05 (one-way ANOVA with Tukey post hoc test).

To determine the effect of L-KYN, KYNA and FICZ on proliferation of human melanocytes and melanoma cells, HEMa, A375 and RPMI7951 cells were exposed to serial dilutions of tested tryptophan-derived AhR ligands. Interestingly, L-KYN and FICZ, but not KYNA exerted antiproliferative activity towards human melanoma A375 and RPMI7951 cells, measured by means of BrdU Assay indicating the level of DNA synthesis (Figure 1). Importantly, the antiproliferative effect was enhanced in A375 cells in comparison to RPMI7951 cells representing metastatic melanoma. L-KYN at a concentration of only 1 pM statistically significantly inhibited cell proliferation of A375 cells, whereas only millimolar concentrations of L-KYN affected proliferation of RPMI7951 cells (Figure 1a). Similarly, A375 cells were more sensitive to the antiproliferative activity of FICZ in comparison to RPMI7951 cells (Figure 1c). However, this compound only moderately inhibited proliferation of melanoma cells by less than 20%. On the other hand, only L-KYN at the highest concentration (5 mM) among selected tryptophan-derived AhR ligands inhibited DNA synthesis in normal human melanocytes HEMa (Figure 1a).

**Figure 2:**
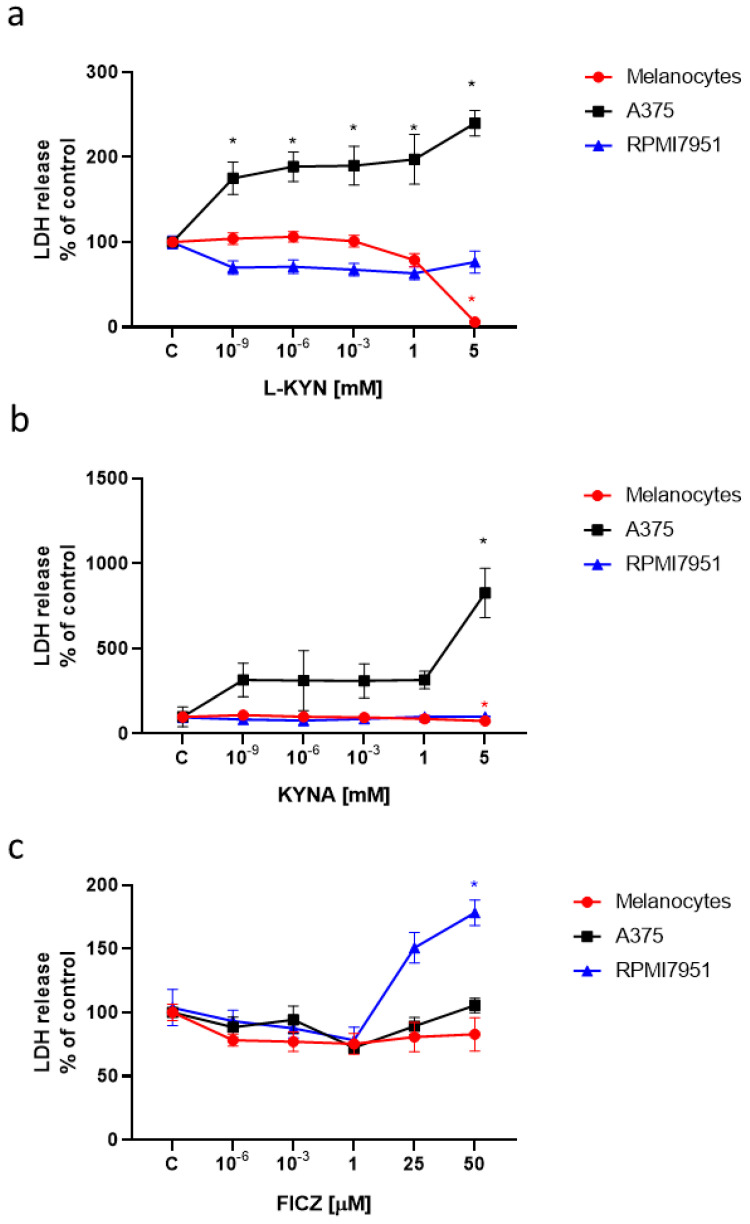
The toxicity of L-KYN (**a**), KYNA (**b**) and FICZ (**c**) towards melanocytes and melanoma A375 and RPMI7951 cells. Normal human adult primary epidermal melanocytes (HEMa) and human melanoma A375 and RPMI7951 cells were exposed to fresh medium (control, C) or serial dilutions of L-KYN, KYNA and FICZ for 24 h. The toxicity of tested compounds was assessed by means of LDH Assay measuring LDH release. Data represent a mean value (% of control) ± SEM of eight independent experiments. Values significant (*) in comparison to control (100%) with *p* < 0.05 (one-way ANOVA with Tukey post hoc test). Positive control for melanoma A375 cells (Total LDH) = 1720%.

Moreover, we tested the toxicity of L-KYN, KYNA and FICZ on human melanocytes and melanoma cells by means of LDH Assay (Figure 2). All tested tryptophan–derived AhR ligands did not induce LDH release and were not toxic to normal melanocytes HEMa. L-KYN and 5 mM KYNA increased LDH release in A375 cells (Figure 2a,b), whereas a toxic effect of FICZ was observed in RPMI7951 cells (Figure 2c).

**Figure 3:**
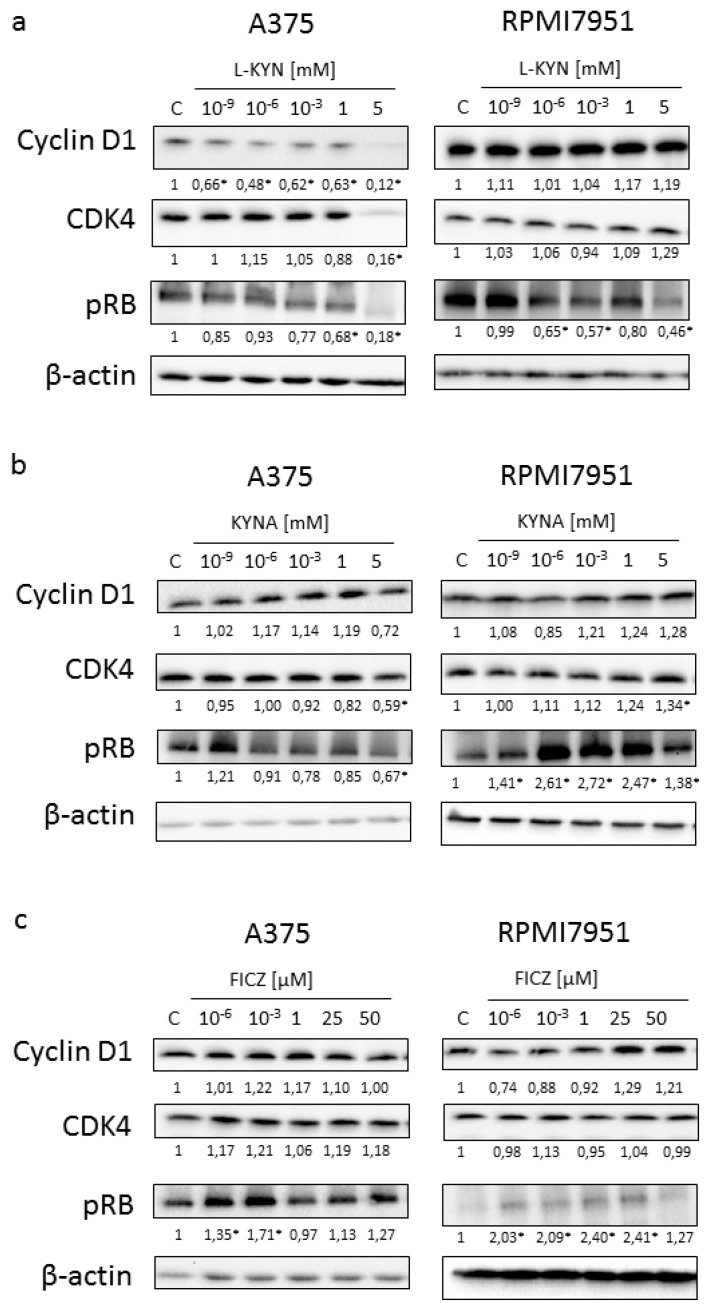
The effect of L-KYN (**a**), KYNA (**b**) and FICZ (**c**) on the protein level of selected cell cycle regulators in melanoma A375 and RPMI7951 cells. Western blot analysis of the protein level of cyclin D1, CDK4 and phosphorylation of Rb in A375 and RPMI7951 cells after treatment with L-KYN (**a**) and KYNA (**b**) in the range of concentrations 10^−9^–5 mM and FICZ (**c**) in the range of concentrations 10^−6^–50 µM for 24 h (C control; not treated). Western blots shown in the figure were selected as the most representative of the series of repetitions. The data were normalized relative to β-actin. The results of densitometric analysis are shown as % of control (the changes ≥30% were considered as significant (*)).

To reveal the potential molecular mechanism of biological activity of selected tryptophan-derived AhR ligands in melanoma cells, the effect of L-KYN, KYNA and FICZ on activation and protein level of selected cell cycle regulators was determined by means of western blot (Figure 3). Similarly to results obtained from BrdU and LDH Assays, we reported the differences in the activation and level of selected proteins in melanoma A375 and RPMI7951 cells, representing successive stages of carcinogenesis.

**Figure 4:**
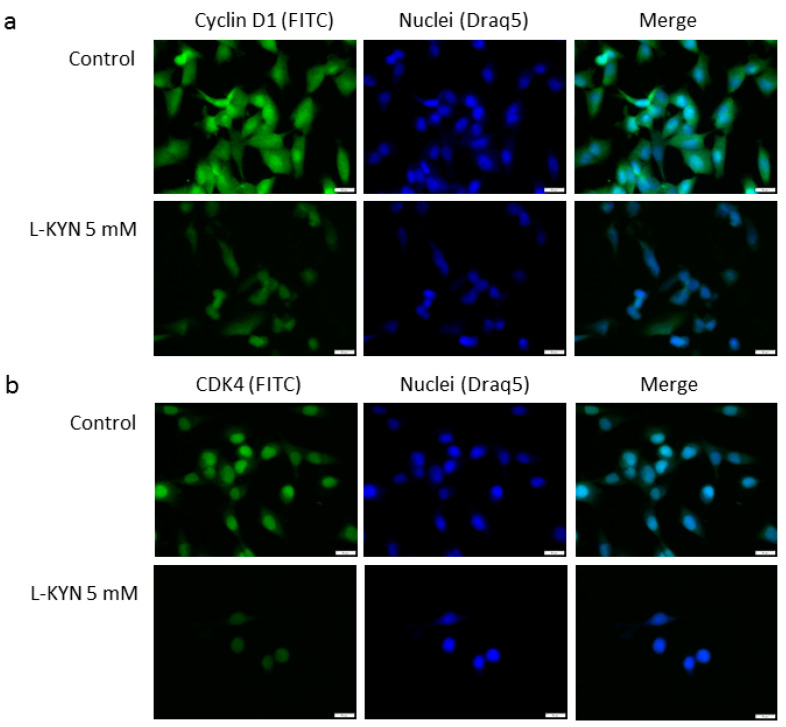
The effect of L-KYN on the protein level and cellular localization of cyclin D1 (**a**) and CDK4 (**b**) in melanoma A375 cells. Immunofluorescent staining of cyclin D1 (**a**) and CDK4 (**b**) in A375 cells treated with L-KYN 5 mM for 24 h (control; not treated). Cell nuclei were labeled with cell permeable fluorescent DNA dye DraQ5. Magnification 40×.

L-KYN inhibited the protein level of cyclin D1 and cyclin-dependent kinase 4 (CDK4) in A375 cells, however, this effect was not observed in RPMI7951 cells (Figure 3a). Immunofluorescence staining confirmed inhibition of cyclin D1 and CDK4 in melanoma A375 cells exposed to L-KYN (Figure 4). No significant cellular relocalisation of cyclin D1 and CDK4 was observed (Figure 4). Moreover, L-KYN decreased phosphorylation of Rb in both A375 and RPMI7951 cells (Figure 3a). KYNA at a concentration of 5 mM significantly decreased the protein level of CDK4 in A375 cells, whereas it increased the protein level of this cell cycle regulator in RPMI7951 cells (Figure 3b). A similar effect was observed in Rb phosphorylation and the protein level of cyclin D1, but these moderate changes in cyclin D1 level were not significant (Figure 3b). Interestingly, we did not observe significant changes in the protein level of cyclin D1 and CDK4 in melanoma A375 and RPMI7951 cells exposed to FICZ (Figure 3c). However, this tryptophan-derived AhR ligand enhanced phosphorylation of Rb (Figure 3c).

**Figure 5:**
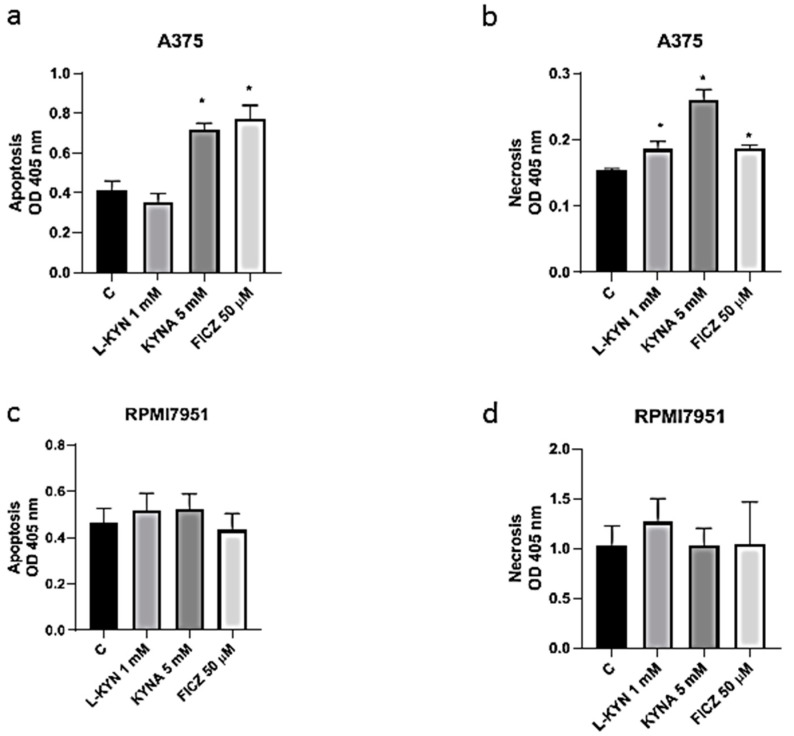
The effect of L-KYN, KYNA and FICZ on induction of apoptosis and necrosis in melanoma A375 (**a**,**b**) and RPMI7951 (**c**,**d**) cells. Human melanoma A375 and RPMI7951 cells were exposed to fresh medium (control, C) or selected tryptophan-derived AhR ligands: L-KYN (1 mM), KYNA (5 mM) and FICZ (50 µM) for 24 h. The effect of tested compounds on induction of apoptosis (**a**,**c**) and necrotic cell death (**b**,**d**) was determined by means of Cell Death Detection ELISA. Data represent a mean value ± SEM of three independent experiments. Values significant (*) in comparison to control with *p* < 0.05 (one-way ANOVA with Tukey post hoc test).

**Figure 6:**
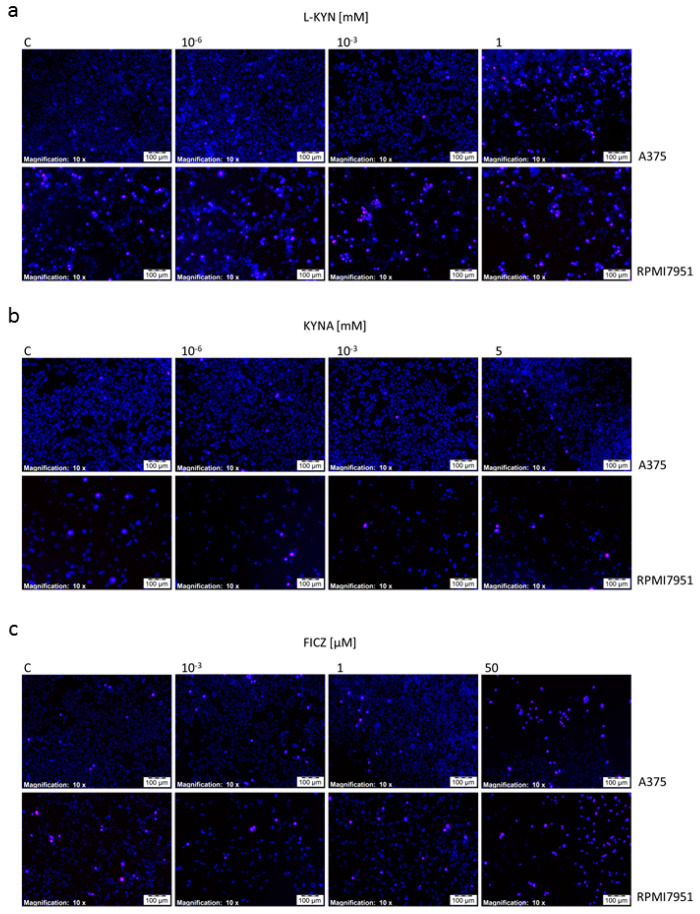
The effect of L-KYN, KYNA and FICZ on induction of cell death in melanoma A375 and RPMI7951 cells. Human melanoma A375 and RPMI7951 cells were exposed to fresh medium (control, C) or selected tryptophan-derived AhR ligands: L-KYN (1 mM) (**a**), KYNA (5 mM) (**b**) and FICZ (50 µM) (**c**) for 24 h. The effect of tested compounds on induction of apoptosis and necrotic cell death was determined by means of staining with Hoechst 33342 and propidium iodide. Cells with fragmented nuclei stained in an intense blue color were considered apoptotic cells while the pink staining of the nuclei was necrotic cells. Magnification 10×.

To investigate the effect of selected tryptophan-derived AhR ligands on induction of cell death in melanoma cells, Cell Death Detection ELISA and fluorescent cell death analysis (Hoechst 33342 and propidium iodide staining) were applied. Taking into consideration the negative effect of 5 mM L-KYN on proliferation of HEMa cells (Figure 1a), this concentration was excluded from further analysis. KYNA 5 mM and FICZ 50 µM statistically significantly induced apoptosis, whereas all tested compounds in higher concentrations (L-KYN 1 mM, KYNA 5 mM, FICZ 50 µM) increased necrosis in melanoma A375 cells (Figure 5a,b). The effect was visualized by Hoechst 33342 and propidium iodide staining (Figure 6). On the other hand, we did not observed statistically significant changes in apoptosis and necrosis induction by selected tryptophan-derived AhR-ligands in melanoma RPMI7951 cells (Figure 5c,d; Figure 6).

**Figure 7:**
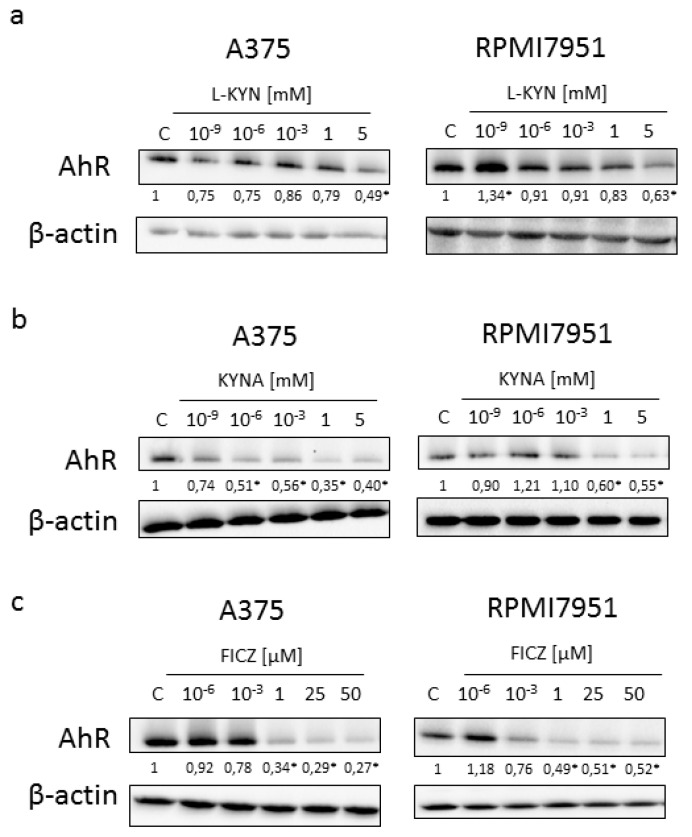
The effect of L-KYN, KYN A and FICZ on AhR protein level in melanoma A375 and RPMI7951 cells. Western blot analysis of protein level of AhR in A375 and RPMI7951 cells after treatment with L-KYN (**a**) and KYNA (**b**) in the range of concentrations 10^−9^–5 mM and FICZ (**c**) in the range of concentrations 10^−6^–50 µM for 24 h (C control; not treated). Western blots shown in the figure were selected as the most representative of the series of repetitions. The data were normalized relative to β-actin. The results of densitometric analysis are shown as % of control (changes ≥30% were considered as significant (*)).

**Figure 8:**
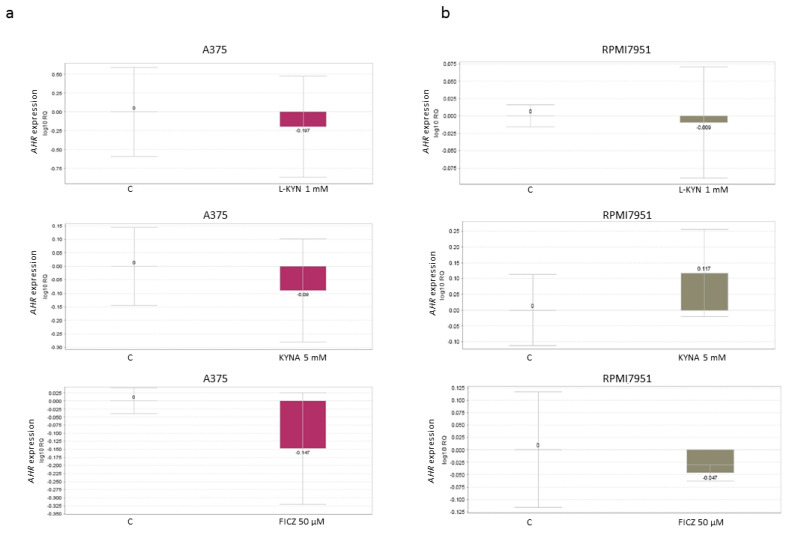
The effect of L-KYN, KYNA and FICZ on *AHR* gene expression in melanoma A375 (**a**) and RPMI7951 (**b**) cells. Real-Time PCR analyses were used to evaluate *AHR* gene expression in A375 and RPMI7951 cells after 24 h treatment with L-KYN, KYNA and FICZ at the concentration of 1mM, 5 mM and 50 µM, respectively (C, control–not treated). *ACTB* was used as the reference gene. For more reliable results, RQ values were analyzed after log transform to log10RQ. Data represent a mean value ± SEM of three independent experiments. The differences in AHR gene expression were not statistically significant in comparison to control (C) (values significant with *p* < 0.05, unpaired *t*-test).

Because all tested compounds are tryptophan-derived AhR ligands, we decided to study the effect of L-KYN, KYNA and FICZ on the expression of AhR. Interestingly, all tested compounds in higher concentrations decreased the protein level of AhR in both melanoma cell lines (Figure 7). However, L-KYN 1 mM, KYNA 5 mM and FICZ 50 µM did not affect the gene expression of *AHR* (Figure 8) in melanoma A375 and RPMI7951 cells.

## 3. Discussion

In this study, we showed that tryptophan-derived AhR ligands, including KYN, KYNA and FICZ, do not stimulate promotion and progression of melanoma in vitro, but simultaneously the tested compounds in higher concentrations inhibited proliferation and stimulated cell death of melanoma cells. This appears to be a priority as the skin is constantly exposed to KYN, KYNA and FICZ belonging to AhR ligands, and the activation of this receptor may be involved in several cellular processes including proliferation, migration or cell death [5,6]. KYN and KYNA are endogenously produced tryptophan metabolites present in various tissues and physiological fluids (reviewed in [15,27,28]). Additionally, previous studies revealed that several food products, herbs and beverages contain KYN [29,30,31,32] and KYNA [16,17,18,33,34], which may be considered as exogenous sources of these tryptophan metabolites. Taking into consideration the increasing use of plant extracts and herbs in cosmetics and skin care products, it can be assumed that the skin is also exposed frequently to certain amounts of KYN and KYNA. On the other hand, FICZ is considered as a photometabolite of tryptophan synthetized in skin after exposure to UV or visible irradiation [22], thus skin is constantly exposed to this compound.

The study was conducted on normal human adult melanocytes and two melanoma cell lines A375 and RPMI7951 differing from each other not only in origin but also in mutated genes. The A375 cell line represents primary malignant melanoma, whereas the RPMI7951 cell line was derived from lymph node metastasis and was reported as a strongly invasive melanoma. Both A375 and RPMI7951 melanoma cells bear the *BRAF* mutation (p.V600E) and *CDKN2A* mutation, however, only A375 cells harbor a homozygous mutation in the *BRAF* gene. Additionally, a mutant *TP53* and *PTEN* were identified only in RPMI7951 cells ([35]; product information ATCC). Thus, the obtained results make it possible to analyze the biological effect of KYN, KYNA and FICZ on melanoma cells representing the subsequent stages of the disease. However, at this stage of research, we are not able to clearly determine whether the differences in the effects of substances result from the stage of the disease or a specific gene mutation.

All selected tryptophan-derived compounds are considered as AhR ligands. AhR plays a role in various physiological and pathological processes in skin, including carcinogenesis [5,6]. Importantly, in the study we confirmed that exposure to KYN, KYNA and FICZ did not stimulate proliferation of melanoma A375 and RPMI7951 cells. On the contrary, KYN and FICZ inhibited DNA synthesis of melanoma cells (Figure 1a,c). Interestingly, more potent inhibitory activity was observed towards melanoma A375 cells (Figure 1). Antiproliferative activity of KYN towards A375 cells was already reported at a concentration of 10^−9^ mM, whereas KYN inhibited proliferation of RPMI7951 cells at a concentration of 5 mM (Figure 1a). Similarly, FICZ statistically significantly inhibited DNA synthesis in A375 cells at a concentration range of 10^−6^–50 μM, but the inhibitory effect towards RPMI7951 cells was observed only in the highest tested micromolar concentrations. These results confirm that RPMI7951 cells, derived from melanoma node metastasis, are strongly invasive and more resistant to pharmacological treatment [36]. Importantly, KYN, KYNA and FICZ did not affect proliferation of normal human melanocytes (HEMa) (Figure 1). Only L-KYN in the highest concentration of 5 mM caused a significant inhibition of DNA synthesis in HEMa cells, therefore this dose was excluded from further studies. Surprisingly, KYNA with evidenced antiproliferative activity towards several cancer cell lines [37,38,39,40,41] did not affect DNA synthesis in melanoma A375 and RPMI7951 cells (Figure 1b). On the other hand, previous studies reported a more potent antiproliferative effect measured by means of MTT Assay, rather than BrdU Assay [38,40,41]. The difference may result from the mechanism of biological activity of KYNA, as the MTT Assay determines metabolic activity of cells, whereas BrdU Assay determines DNA synthesis. Similarly, despite no changes in DNA synthesis in KYNA-treated A375 cells (Figure 1b), the cytotoxicity assay confirmed statistically significant increase in LDH release in these cells (Figure 2b), which may suggest a different mechanism of KYNA biological activity towards melanoma cells. A reduction of LDH release in melanocytes exposed to KYN 5 mM probably results from the strong antiproliferative effect of the compound confirmed in the BrdU Assay (Figure 2b). In conclusion, the presented results showed that KYNA, KYNA and FICZ, despite belonging to the same group of tryptophan-derived AhR ligands, showed different mechanisms of action.

This hypothesis was also confirmed in the molecular studies. The dysregulation of the p16-cyclinD-CDK4/6-Rb pathway is common in melanomas and occurs in 22–78% of cases [42]. Thus, we studied the effect of KYN, KYNA and FICZ on the protein level of cyclin D1, CDK4 and Rb phosphorylation in melanoma A375 and RPMI7951 cells (Figure 3). Cyclin D1, CDK4 and Rb control the G1/S transmission. Previous studies revealed the positive correlation between elevated CDK4 expression and increased therapeutic activity of CDK4/6 inhibitors undergoing clinical trials or currently used in anti-cancer therapy [42,43,44,45]. Our studies revealed that KYN inhibited the protein level of cyclin D1 and CDK4 in melanoma A375 cells, but not in more resistant RPMI7951 cells (Figure 3a). However, KYN affected the activation of Rb, one of the key regulators of cell cycle in both melanoma cell lines (Figure 3a). Interestingly, KYNA in the highest tested concentration (5 mM) decreased CDK4 protein level and phosphorylation of Rb in A375 cells (Figure 3b), despite no significant changes in proliferation (DNA synthesis) of KYNA-treated A375 melanoma cells. However, it cannot be excluded that the biological effect of the observed molecular changes in cell cycle regulators would be seen after a longer incubation time. The opposite effect of KYNA on the protein level and activation of cell cycle regulators was observed in RPMI7951 cells. Surprisingly, despite the increased phosphorylation of Rb in RPMI7951 cells exposed to KYNA (Figure 3b), no significant changes in proliferation was observed (Figure 1b). Similarly, enhanced phosphorylation of Rb was determined in A375 and RPMI7951 cells exposed to FICZ (Figure 3c). Although the exact biological effect of the Rb activity in melanoma A375 and RPMI7951 cells exposed to FICZ and RPMI8951 cells exposed to KYNA is unknown, it should be noted that Rb interacts not only with cyclins, CDKs, phosphatases and chromatin-associated proteins but is also involved in regulation of metabolic pathways [46,47,48]. Previous studies revealed that *RB1* mutant cells are characterized by reduced mitochondrial respiration, polarity and alternation with metabolic flux (reviewed in [46,48]). Bearing in mind the origin of these AhR ligands as tryptophan metabolites, it cannot be excluded that phosphorylation of Rb may be associated with metabolic changes in A375 and RPMI7951 cells exposed to KYNA and FICZ. Importantly, previous studies suggested a relationship between cellular metabolism and the anti-tumor activity of KYNA [15].

Additionally, we also revealed that tryptophan-derived AhR ligands affect not only proliferation of melanoma cells in vitro, but may also induce cell death in melanoma A375 cells (Figure 5 and Figure 6). KYN, KYNA and FICZ increased necrosis in A375 cells, but a significant increase in apoptotic cells was determined only in A375 cells exposed to KYNA and FICZ. Interestingly, no positive or negative effect on cell death was observed in RPMI7951, representing a more resistant melanoma cell line (Figure 3). However, it cannot be excluded that the differences in the effects of selected tryptophan metabolites in A375 and RPMI7951 cells result from a specific gene mutation in tested cell lines. It should be noted that RPMI7951 cells harbor a *TP53* mutation, thus the crucial role of p53 in the induction of apoptosis in A375 cells may be suggested and needs further investigation.

KYN, KYNA and FICZ represent tryptophan-derived AhR ligands, so we decided to investigate their effect on the protein and gene expression of AhR. Surprisingly, although all tested compounds decreased the protein level of AhR in a dose-dependent manner, none of the tryptophan-derived AhR ligands affected gene expression of *AHR* (Figure 7 and Figure 8). These results suggest that KYN, KYNA and FICZ may increase the proteolytic degradation of AhR but not interfere at the genome level. Similar results were reported previously by Mengoni et al. [7] in a panel of melanocytic and dedifferentiated human melanoma cell lines treated with FICZ. This mechanism may be a form of a negative regulation of AhR activity in melanoma cells protecting against excessive activity of AhR-dependent pathways. On the other hand, it cannot be excluded that it is one of the mechanisms of biological activity of tested tryptophan-derived AhR ligands leading to the inhibition of proliferation, increased toxicity and increased death of cancer cells.

Despite the common origin of KYN, KYNA and FICZ as tryptophan metabolites, the activity of these substances on melanoma cells and their molecular mechanism of biological activity are different. Moreover, due to similarity of the activity of all tested tryptophan metabolites towards AhR, and the differences in the biological effects of their activity on proliferation, cell cycle regulation and cell death, it may be suggested that the molecular mechanism of KYN, KYNA and FICZ is not only dependent on AhR. Although further studies should be conducted to reveal the specific mechanism of activity of tested tryptophan metabolites towards melanoma cells, taking into consideration the obtained results, we may conclude that KYN, KYNA and FICZ do not promote melanoma A375 and RPMI7951 cell proliferation and growth in vitro. However, to exclude the pro-carcinogenic potential of tryptophan-derived AhR ligands, further in vivo and clinical trials are necessary. Despite the undisputed value of the results showing the biological activity of KYN, KYNA and FICZ towards melanoma cells, in vitro studies do not take into account the influence of the tested substances on the immune system and the additional influence of KYN and KYNA from food products. It should be noted that KYN is strongly associated with immunosuppression and cancer escape from immune surveillance [28]. Moreover, in order to exclude possible negative effects of frequent exposure to tryptophan-derived AhR ligands on the skin, further research into effects on other skin cells should also be performed.

In conclusion, the selected tryptophan-derived AhR ligands, KYN, KYNA and FICZ, produced endogenously in the skin and present in herbs and plant extracts used in skin care treatments, did not promote melanoma cell growth in vitro, and even in higher concentrations they may exhibit antiproliferative and cytotoxic activity and promote cell death in melanoma A375 and RPMI7951 cells. However, the biological activity and molecular mechanism was different for each compound and not strictly dependent on AhR.

## 4. Materials and Methods

### 4.1. Drugs

L-KYN and KYNA were obtained from Sigma Aldrich (St. Louis, MO, USA). L-KYN was dissolved in cell culture medium, whereas KYNA was dissolved in 1 N NaOH, and then phosphate buffered saline (PBS). FICZ, obtained from Tocris Bioscience (Bristol, UK), was dissolved in dimethyl sulfoxide (DMSO). The final concentration of DMSO in cell culture medium dilutions was less than 0.2%. No significant effects of solvents on melanoma and melanocyte cell proliferation and morphology were observed.

### 4.2. Cell Cultures

Normal human adult primary epidermal melanocytes (HEMa) and human melanoma A375 and RPMI7951 cells were obtained from American Type Culture Collection (ATCC; Manassas, VA, USA). HEMa cells were cultured in Dermal Cell Basal Medium supplemented with Adult Melanocyte Growth Kit (ATCC; Manassas, VA, USA). A375 cells were grown in Dulbecco’s modified Eagle’s medium (DMEM) supplemented with 10% heat inactivated fetal bovine serum (FBS), penicillin (100 U/mL) and streptomycin (100 µg/mL). RPMI7951 cells were grown in Minimum Essential Medium with Earle′s salts supplemented with sodium pyruvate (final concentration 1 mM), 10% heat inactivated FBS, penicillin (100 U/mL) and streptomycin (100 µg/mL). All A375 and RPMI 7951 cell culture reagents were purchased from Sigma Aldrich (St. Louis, MO, USA). Cells were maintained in a humidified atmosphere of 95% air and 5% CO_2_ at 37 °C.

### 4.3. Experiment Design

Previous studies revealed that KYN competes for a membrane transporter with tryptophan [49]. To exclude any interactions between tryptophan-derived AhR ligands and tryptophan included in the culture medium and to provide the maximum of bioavailability of the tested compounds, melanocytes or melanoma cells (~60–70% confluence) were treated with serial dilutions of L-KYN, KYNA and FICZ dissolved in Hanks’ Balanced Salt solution (HBSS, Sigma Aldrich, St. Louis, MO, USA) for 1 h in standard conditions. After incubation time, HBSS was discarded, and cells were exposed to fresh medium (control) or serial dilutions of L-KYN, KYNA or FICZ in a fresh medium and incubated for 23 h in standard conditions. Then further analyses were performed (LDH assay, BrdU assay, Cell Death Detection ELISA, western blot, PI and Hoechst staining, RT-PCR).

### 4.4. BrdU Assay

BrdU assay, quantifying the incorporation of 5′-bromo-2′-deoxy-uridine (BrdU) into newly synthesized DNA of actively proliferating cells, was used to determine the effect of L-KYN, KYNA and FICZ on proliferation of melanocyte and melanoma cells according to the procedure described previously [38]. Briefly, HEMa, A375 and RPMI7951 cells were plated in 96-well plates (NUNC, Roskilde, Denmark) at the density of 4 × 10^4^ cells/mL, 2 × 10^4^ cells/mL and 4 × 10^4^ cells/mL, respectively. Next day, the cells were exposed to serial dilutions of the tested compound (L-KYN: 10^−9^, 10^−6^, 10^−3^, 1, 5 mM; KYNA: 10^−9^, 10^−6^, 10^−3^, 1, 5 mM, FICZ: 10^−6^, 10^−3^, 1, 25, 50 µM) or fresh cell culture medium (control, C) according to the experiment design described in detail above. Cell proliferation was quantified after 24 h incubation according to the manufacturer’s procedure (Cell Proliferation ELISA BrdU, Roche Diagnostics GmbH, Penzberg, Germany).

### 4.5. LDH Assay

The In Vitro Toxicology Assay Kit (TOX-7) (Sigma Aldrich, St. Louis, MO, USA), based on the reduction of NAD by lactic dehydrogenase (LDH) released from damaged cells, was applied to determine the cytotoxicity of L-KYN, KYNA and FICZ. HEMa, A375 and RPMI7951 cells were plated in 96-well plates (NUNC, Roskilde, Denmark) at the density of 4 × 10^4^ cells/mL, 2 × 10^4^ cells/mL and 4 × 10^4^ cells/mL, respectively. The next day, the cells were exposed to serial dilutions of tested compound (L-KYN: 10^−9^, 10^−6^, 10^−3^, 1, 5 mM; KYNA: 10^−9^, 10^−6^, 10^−3^, 1, 5 mM, FICZ: 10^−6^, 10^−3^, 1, 25, 50 µM) or fresh cell culture medium (control, C) according to the experiment design described in detail above. The activity of released LDH in the supernatants was measured after 24 h incubation according to the manufacturer’s procedure and was quantified at 490 nm (Epoch microplate reader (BioTek Instruments, Inc., Winooski, VT, USA) equipped with Gen5 software (v. 2.01, BioTek Instruments, Inc., Winooski, VT, USA)).

### 4.6. Western Blot

Melanoma A375 and RPMI7951 cells were exposed to serial dilutions of the tested compounds (L-KYN: 10^−9^, 10^−6^, 10^−3^, 1, 5 mM; KYNA: 10^−9^, 10^−6^, 10^−3^, 1, 5 mM, FICZ: 10^−6^, 10^−3^, 1, 25, 50 µM) or fresh cell culture medium (control, C) for 24 h according to the experiment design described in detail above. The protein level or its activation was determined by means of western blot procedure previously described in [50]. The following primary antibodies were used in the procedure: cyclin D1, CDK4, phospho-Rb (Ser 807/811), AhR and β-actin antibody (1:1000; Cell Signaling Technology, Danvers, MA, USA). In the procedure the secondary antibodies coupled to horseradish peroxidase were used (1:2000) (Cell Signaling Technology, Danvers, MA, USA). The blots were visualized by using enhanced chemiluminescence (Pierce Biotechnology, Waltham, MA, USA) and the Syngene G:BOX Chemi XT4 gel documentation system (Syngene, Cambridge, UK).

### 4.7. Immunofluorescence Staining

A375 cells cultured on LabTek Chamber Slides (Nunc, ThermoFisher Scientific, Roskilde, Denmark) were exposed to culture medium (control, C) or L-KYN 5 mM for 24 h in standard conditions according to the experiment design described in detail above. Then, cells were fixed with 3.7% paraformaldehyde, permeabilized with 0.2% Triton X-100, and treated with 5% bovine serum albumin (BSA, Sigma Aldrich, St. Louis, MO, USA). The cells were exposed to primary antibodies against cyclin D1 and CDK4 (1:100; Cell Signaling Technology, Danvers, MA, USA) overnight at 4 °C and then were incubated with secondary antibodies conjugated with fluorescein isothiocyanate (FITC) (1:100) (Sigma Aldrich, St. Louis, MO, USA) for 2 h at room temperature. Cell nuclei were labeled with cell permeable fluorescent DNA dye DraQ5 (Cell Signaling Technology, Danvers, MA, USA). Cell images were captured with fluorescence microscopy (automatic microscope Olympus IX83; Olympus Optical Co., Ltd., Tokyo, Japan, and CellSens RT software, Olympus Optical Co., Ltd., Tokyo, Japan) at 40× magnification.

### 4.8. Cell Death Detection ELISA

The effect of the tryptophan-derived AhR ligands, L-KYN, KYNA and FICZ, on apoptosis and necrosis of A375 and RPMI7951 melanoma cells was assessed by ELISA. The Cell Death Detection ELISA^PLUS^ photometric enzyme immunoassay was used for the quantitative in vitro determination of cytoplasmic histone-associated DNA fragments (mono- and oligonucleosomes) after induced cell death. A375 and RPMI7951 melanoma cells were plated in 96-well plates (NUNC, Roskilde, Denmark) at the density of 2 × 10^4^ cells/mL and 4 × 10^4^ cells/mL, respectively. Next day, the cells were exposed for 24 h to serial dilutions of tested compound (L-KYN 1 mM, KYNA 5 mM, FICZ 50 µM) or fresh cell culture medium (control, C) according to the experiment design described in detail above. ELISA test was performed according to the manufacturer’s procedure. The colorful product was quantified spectrophotometrically at 405 nm using a microplate reader (Epoch, BioTek Instruments, Inc., Winooski, VT, USA) equipped with Gen5 software (v. 2.01, BioTek Instruments, Inc., Winooski, VT, USA).

### 4.9. Fluorescent Cell Death Analysis—Hoechst 33342 and Propidium Iodide Staining

Melanoma A375 and RPMI7951 cells were plated on Lab-Tek Chamber Slides (NUNC, Roskilde, Denmark) at the density of 4 × 10^4^ cells/mL and 8 × 10^4^ cells/mL, respectively. The next day, cells were exposed to serial dilutions of tested compound (L-KYN: 10^−6^, 10^−3^, 1 mM; KYNA: 10^−6^, 10^−3^, 5 mM, FICZ: 10^−3^, 1, 50 µM) or fresh cell culture medium (control, C) according to the experiment design described in detail above. After 24 h treatment, the effect of L-KYN, KYNA and FICZ on induction of cell death was analyzed after fluorescence staining with Hoechst 33342 and propidium iodide at a concentration of 0.2 μg/mL and 0.4 μg/mL, respectively (5 min at 37 °C). Cell images were captured with fluorescence microscopy (Olympus IX83 System Microscope; Olympus Optical Co., Ltd. and CellSens RT software, Olympus Optical Co., Ltd., Tokyo, Japan) at 10× magnification.

### 4.10. Real-Time PCR

Melanoma A375 and RPMI7951 cells were exposed to the tested compounds (L-KYN 1 mM, KYNA 5 mM, FICZ 50 µM) or fresh cell culture medium (control, C) for 24 h according to the experiment design described in detail above. Extracted total RNA (High Pure RNA Isolation Kit (Roche Diagnostics GmbH, Penzberg, Germany)) was reverse-transcribed using a High Capacity cDNA Reverse Transcription Kit (Applied Biosystems, Foster City, CA, USA) according to the manufacturer’s procedure. Real-time PCR analyses were performed using TaqMan Gene Expression Assays (IDs: Hs00169233_m1 for *AHR* and Hs99999903_m1 for reference gene *ACTB*; ThermoFisher Scientific, Waltham, MA, USA) and TaqMan Fast Universal PCR MasterMix (ThermoFisher Scientific, Waltham, MA, USA) as previously described [51] using a QuantStudio 12K Flex (Appllied Biosystems, Foster City, CA, USA). The expression of reference gene *ACTB* was used as an endogenous control. The relative expression was calculated by the formula RQ = 2*^–^*^ΔΔ*C*t^ [52] (QuantStudioTM 12K Flex Softwere v1.2.2, Applied Biosystems, Foster City, CA, USA).

### 4.11. Data Analysis

The data were plotted as the mean value ± standard error of the mean (SEM) and analyzed by means of GraphPad Prism 8 software (GraphPad Software, Inc., La Jolla, CA, USA). Statistical analysis was performed using one-way ANOVA with Tukey post hoc test or unpaired *t*-test (significance was accepted at *p* < 0.05). The western blots shown in the figures were selected as the most representative among repetitions *n* ≥ 3. Western blots were quantified densitometrically using NIH ImageJ software (Wayne Rasband, Bethesda, MD, USA) and are shown as relative value of control (the fold changes in protein level ≥30% were considered as significant; qualitative analysis). The data were normalized relative to β-actin.

## Abbreviations

AhR	aryl hydrocarbon receptor
BSA	bovine serum albumin
BrdU	5′-bromo-2′-deoxy-uridine
CDK	cyclin-dependent kinase
DMEM	Dulbecco’s modified Eagle’s medium
DMSO	dimethyl sulfoxide
FBS	fetal bovine serum
FICZ	6-formylindolo[3,2-b]carbazole
FITC	fluorescein isothiocyanate
HBSS	Hanks’ Balanced Salt solution
HEMa	normal human adult primary epidermal melanocytes
IDO	indoleamine 2,3-dioxygenase
KYNA	kynurenic acid
KYN	kynurenine
LDH	lactic dehydrogenase
PBS	phosphate buffered saline
Rb	retinoblastoma tumor suppressor protein
SEM	standard error of the mean
TDO	tryptophan 2,3-dioxygenase
